# Evaluating posterior vitreous detachment by widefield 23-mm swept-source optical coherence tomography imaging in healthy subjects

**DOI:** 10.1038/s41598-021-99372-z

**Published:** 2021-10-05

**Authors:** Yoshiaki Chiku, Takao Hirano, Yoshiaki Takahashi, Ayako Tuchiya, Marie Nakamura, Toshinori Murata

**Affiliations:** Department of Ophthalmology, Shinshu University School of Medicine, 3-1-1 Asahi, Matsumoto, Nagano 390-8621 Japan

**Keywords:** Diseases, Medical research

## Abstract

Posterior vitreous detachment (PVD) is a separation between the posterior vitreous cortex and internal limiting membrane. Although PVD was historically considered an acute event, recent studies using spectral-domain optical coherence tomography (SD-OCT) revealed a gradual progression of PVD in healthy subjects. Although SD-OCT improved PVD studies, the narrow imaging angle and long examination time were problematic to allow wide angle capture. The Xephilio OCT-S1 (Canon), a swept-source OCT (SS-OCT) device, can obtain up to 23-mm of widefield B-scan images in a single acquisition. We used this widefield SS-OCT to quantitatively evaluate the PVD stage in 214 healthy subjects aged 4–89 years and determine whether PVD stages differ between the bilateral eyes of each patient. Age was significantly positively correlated with the overall PVD stage (ρ = 0.7520, *P* < 0.001). Interestingly, partial PVD occurred in children as young as 5 years, indicating that initial PVD onset may occur much earlier than previously reported. Furthermore, PVD stages of the bilateral eyes were highly consistent in 183 subjects (85.5%). Widefield 23-mm SS-OCT thus revealed that PVD started earlier than anticipated, and age was correlated with the symmetry of PVD stage. Widefield 23-mm SS-OCT may also be clinically useful for the evaluation of diseased eyes.

## Introduction

Posterior vitreous detachment (PVD) is a separation between the posterior vitreous cortex and the internal limiting membrane caused by vitreous gel liquefaction and weakening of vitreoretinal adhesion^1^. Although PVD progresses gradually with age in healthy subjects, it can often lead to the development of rhegmatogenous retinal detachment^2^, macular hole^3^, epiretinal membrane^4^, vitreomacular traction syndrome^5^, and other ocular conditions. Historically, PVD was thought to be a sudden and rapidly evolving event because patients with vitreopapillary separation presented with acute-onset flashes and floaters. However, recent studies using spectral-domain optical coherence tomography (SD-OCT) have revealed the gradual and slow progression of PVD in healthy subjects, starting from the paramacular area, extending to the perifoveal area, followed by vitreopapillary separation, and finally leading to complete PVD^6,7^. Although SD-OCT has improved PVD studies, it remains difficult to obtain a wide angle from the optic nerve papilla to the macula and a deep range from the retina to the vitreous cavity containing the posterior precortical vitreous pocket. Montaged OCT images, which are composed of three OCT images merged together, have been created to address these issues^8^. However, this montage-based method requires additional time to obtain multiple scans (with overlapping areas) and has inaccuracies due to subtle misalignments. Therefore, this method can be challenging in children who could not maintain a fixed position.

The recently developed swept-source OCT (SS-OCT) uses a long wavelength of approximately 1000 µm, enabling deeper penetration. Therefore, SS-OCT can acquire images with a deeper range in a single acquisition. Additionally, the scan rate in commercially available SS-OCTs is nearly two-fold faster than in conventional SD-OCTs. This high scan speed reduces motion artifacts and enables acquisition of widefield B-scan images^9^. The Xephilio OCT-S1 (Canon, Tokyo, Japan), launched in 2020, is an SS-OCT device that can obtain up to 23 mm of widefield B-scan images in a single acquisition. This instrument can detect a wide angle of vitreoretinal interfaces for an accurate assessment of PVD progression in a wide population, including young children. However, previous studies on PVD using OCT lacked quantitative assessments and included a possibility of left–right eye differences in PVD progression.

The PVD stage is determined by the location of detachment of the posterior vitreous cortex and the internal limiting membrane. If the location is paramacular, it is defined as stage 1; if it is perifoveal, it is defined as stage 2. After the PVD of macula occurs, it is defined as stage 3 or higher. Paramacular and perifoveal assessments are qualitative assessments, and thus, examiner subjectivity may affect the results. As such, a more quantitative assessment is preferred. In addition, reports on left–right differences in PVD stage are scarce. In most healthy individuals, both eyes are not anatomically and functionally identical, but they generally appear to be similar. If there is a change in interocular symmetry, the physician should establish whether it is due to the disease or constitutes asymmetry within the normal range, as this can influence the treatment plan. Understanding interocular symmetry can be an important factor in the diagnosis, treatment, and follow-up of various diseases.

The current study aimed to quantitatively evaluate the PVD stage in healthy subjects, including infants, using widefield SS-OCT. In addition, we assessed the PVD stage in the bilateral eyes.

## Results

**Figure 1:**
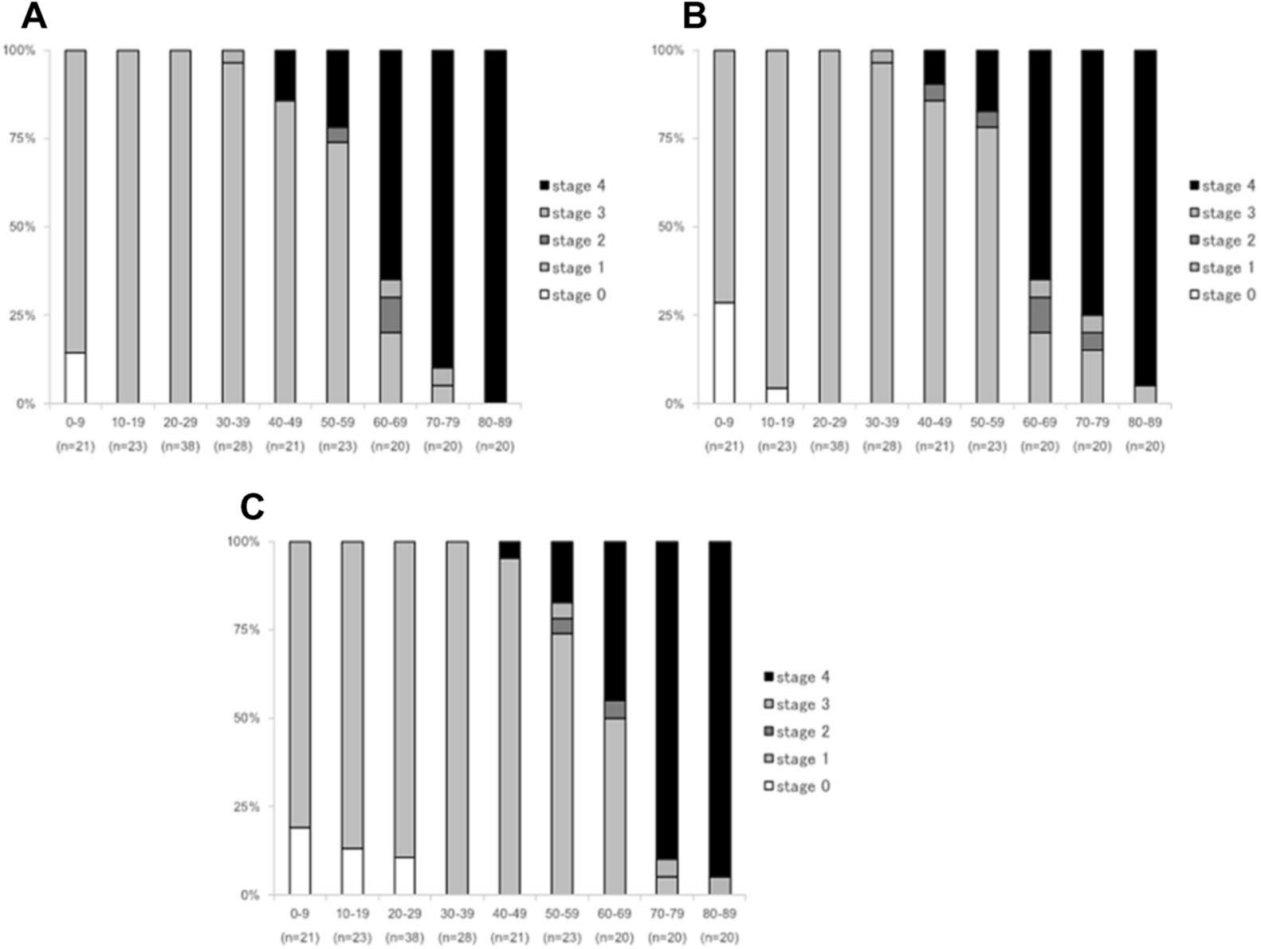
Proportion of subjects in each stage of posterior vitreous detachment by decade of life. Percentages for each stage of PVD ((**A**) eyes with more advanced PVD stage, (**B**) right eyes, (**C**) left eyes) are displayed by age group.

A total of 214 subjects (124 females, 90 males) with a mean age of 42.1 ± 24.4 years (range: 4–89 years) were evaluated. Table 1 presents the clinicodemographic characteristics of subjects. Overall, three subjects had stage 0 PVD (mean age, 5.3 ± 1.5 years; range, 4–7 years); 146 subjects, stage 1 (mean age, 29.9 ± 15.9 years; range, 5–72 years); three subjects, stage 2 (mean age, 60.3 ± 4.5 years; range, 56–65 years); three subjects, stage 3 (mean age, 62.0 ± 20.4 years; range, 39–78 years); and 59 subjects, stage 4 (mean age, 72.0 ± 10.9 years; range, 44–89 years) (Fig. 1A). There was a significant positive correlation between age and PVD stage (ρ = 0.7520, *P* < 0.001).

**Table 1:** Baseline clinicodemographic characteristics of subjects.

Characteristics	Data
**Age (years)**	
Mean ± standard deviation	42.1 ± 24.4
Range	4–89
**Sex, n (%)**	
Female	124 (57.9%)
Male	90 (42.1%)
**Eye (n, %)**	
Right	214 (50%)
Left	214 (50%)
**Lens status, n (%)**	
Phakic	337 (89.9%)
Pseudophakic	38 (10.1%)
**Refractive error category, n (%)**	
Low myopia (≧ − 5 and < − 1 D)	326 (76.2%)
Emmetropia (≧ − 1 and ≦ + 1 D)	79 (18.5%)
Low hyperopia (> + 1 D and ≦ + 3 D)	23 (5.4%)

Data on age and sex are listed for subjects, and data on lens status and refractive error category are listed for eyes.

*D* diopters.

The PVD stage in the right eye was stage 0 in seven subjects (mean age, 6.9 ± 2.0 years; range, 4–10 years); stage 1 in 146 subjects (mean age, 31.7 ± 16.8 years; range, 5–80 years); stage 2 in five subjects (mean age, 60.2 ± 8.9 years; range, 47–71 years); stage 3 in three subjects (mean age, 62.0 ± 20.4 years; range, 39–78 years); and stage 4 in 53 subjects (mean age, 72.6 ± 10.8 years; range, 44–89 years) (Fig. 1B). There was a significant positive correlation between age and PVD stage in the right eye (ρ = 0.7375, *P* < 0.001).

Meanwhile, the PVD stage in the left eye was stage 0 in 11 subjects (mean age, 13.8 ± 7.8 years; median, range, 4–24 years); stage 1 in 147 subjects (mean age, 32.3 ± 17.0 years; range, 5–72 years); stage 2 in two subjects (mean age, 60.5 ± 6.4 years; range, 56–65 years); stage 3 in three subjects (mean age, 70.7 ± 14.5 years; range, 54–80 years); and stage 4 in 51 subjects (mean age, 73.8 ± 9.8 years; range, 47–89 years) (Fig. 1C). There was also a significant positive correlation between age and PVD stage in the left eye (ρ = 0.7376, *P* < 0.001).

There was no significant difference in PVD progression between the left and right eyes in the same subject (*P* = 0.3567). The PVD stages of the bilateral eyes were highly consistent in 183 subjects (85.5%) (Table 2). However, only zero (0%) and one (0.5%) subject had concordant stages 2 and 3 PVD in the bilateral eyes, respectively.

**Table 2:** Comparison of PVD stages in bilateral eyes.

Right eye	Left eye					
	Stage 0	Stage 1	Stage 2	Stage 3	Stage 4	Total
Stage 0	**3**	4	0	0	0	7
Stage 1	8	**134**	1	0	3	146
Stage 2	0	2	**0**	0	3	5
Stage 3	0	2	0	**1**	0	3
Stage 4	0	5	1	2	**45**	53
Total	11	147	2	3	51	**214**

Data are presented as the number of subjects.

Bold values indicate the number of subjects who had the same PVD stage in bilateral eyes.

*PVD* posterior vitreous detachment.

There were no significant differences in the mean age between males and females (41.9 ± 24.4 vs. 42.2 ± 24.2, *P* = 0.8824) and in the PVD stage between both sexes (2.3 ± 1.5 vs. 2.2 ± 1.5, *P* = 0.7262).

## Discussion

Quantitative assessments of PVD using OCT are scarce. This study found that in eyes without pathologies, partial PVD can be observed even at age ≤ 10 years and that the PVD stage progressed with age. Furthermore, there was no significant difference in the PVD stage between the left and right eyes in the same patient.

PVD was historically considered as an acute event occurring due to an abrupt break in the posterior vitreous cortex leading to mechanical collapse and separation of the degenerated vitreous cortex away from the retina^1^. However, OCT has shown that PVD progression is of more chronic nature rather than an acute event^10,11^. Previous studies using 7-mm and 9-mm time-domain OCTs have reported that partial PVD begins in the 30 s in healthy subjects^6,12^. However, the results of our study showed partial PVD even in children as young as 5 years, indicating that the initial onset of PVD is much earlier than that reported.

This discrepancy could be due to the following reasons: first, the difference in the age of the subjects enrolled. Previous reports have examined healthy adult eyes, and few have examined PVD in normal eyes of minors using OCT^6,8,12^. It is assumed that minors were not enrolled due to the long testing time of OCT in these studies. Given that the SS-OCT device used in this study had a shorter acquisition time than the conventional SD-OCT device or montaged OCT method requiring multiple OCT images, it was possible to enroll minors, including children who have difficulty with prolonged positioning. The second reason is the imaging range. In a study using 25–36-mm OCT images montaged from three OCT images, 34 of 36 healthy subjects in their 20 s had partial PVD^8^. This result suggests that PVD begins at a younger age than previously reported.

Moreover, in a study comparing 6-mm and 16.5-mm OCT images in the same eye, seven eyes were categorized as no PVD on 6-mm OCT, but these were upgraded to partial PVD on 16.5-mm OCT^13^. The study concluded that 6-mm OCT scans cannot detect early partial PVD. Considering these previous findings, we hypothesized that the earlier age onset of stage 1 PVD in our study was because we were able to identify peripheral partial PVD by using widefield OCT images. To confirm this hypothesis, we additionally examined the PVD stage on a 6-mm OCT image centered on the fovea taken from the 23-mm OCT image used in this study. On the 6-mm OCT images, 85.7%, 56.5%, 44.7%, 25.0%, 10.0%, 17.4%, and 5.0% of those aged < 10 years, 10–19 years, 20–29 years, 30–39 years, 40–49 years, 50–59 years, and 60–69 years displayed complete vitreoretinal adhesion in the macula and tended to show no PVD (stage 0). However, these patients were found to have stage 1 partial PVD on the original 23-mm widefield OCT image (Supplemental Figure 1), as shown in the representative case in Supplemental Figure 2.

To minimize the effect of individual subject characteristics on the statistical analysis, SS-OCT images were obtained for bilateral eyes in all subjects, but only the eyes of those with advanced PVD stage were first evaluated. Then, we examined the bilateral eyes and found that the rate of PVD progression was identical in both left and right eyes. There was no significant difference in the PVD stage progression between the left and right eyes. To our best knowledge, this is the first study to evaluate and report that there is no difference in PVD progression between the left and right eyes in the same subject.

Sex differences in the incidence of ocular diseases have been reported, such as a higher incidence of rhegmatogenous retinal detachment in males than in females^14^ and a higher incidence of macular hole in females than in males^15^. However, there have been no reports on the individual incidence of PVD in the left and right eyes. The lack of significant differences in the longitudinal PVD progression between the left and right eyes may explain the similar incidence of PVD-related rhegmatogenous retinal detachment and macular hole between the left and right eyes.

Although the PVD stage was concordant in the two eyes in 80% of the subjects, there were few subjects who had concordant stage 2 or stage 3 PVD. In addition, eight subjects (3.7%) had three different PVD stages, such as stage 1 on the right eye and stage 4 on the left eye. Although the reason for this difference is unclear, it should be noted that assessment of only one eye may lead to under- or overestimation of the PVD stage. In clinical practice, patients with macular hole in one eye should be evaluated for PVD in the other eye, and if that eye does not show fovea detachment, i.e., PVD stage ≤ 2, regular follow-up should be recommended.

Several limitations of this study should be acknowledged. First, it was difficult to assess PVD in patients with vitreoschisis. In a study using scanning electron microscopic observations, 44% of eyes with spontaneous PVD showed vitreous cortex remnants on the retinal surface^16^. A conventional SD-OCT study also detected vitreoschisis in 53.3% of eyes with macular holes and 43.2% of eyes with macular pucker^17^. In a study using montaged OCT, vitreoschisis was prevalent in 41.2% of eyes without vitreous-retina-choroid pathologies^8^. It is difficult to distinguish the vitreoschisis line from the posterior vitreous line. This may explain the higher incidence of stage 1 PVD in this study than in previous reports. SD-OCT uses a shorter OCT wavelength and thus has a higher resolution than SS-OCT^18^. Future research should examine the partial PVD sites in more detail by using SD-OCT. Second, although the vitreous status without the refraction of the vitreous cortex can be classified as either no PVD or complete PVD, the differentiation is difficult, especially using SD-OCT. However, SS-OCT has improved depth visualization with a longer OCT wavelength and faster scanning rate than SD-OCT. High depth resolution has a strong potential to detect floating vitreous after completing PVD. In a previous study, the accuracy of the diagnosis of complete PVD by SS-OCT was compared with the definitive diagnosis of B-scan ultrasonography. It was reported that the definitive diagnosis of complete PVD by SS-OCT and B-scan ultrasonography showed a high agreement rate of 83.2%^19^. Additionally, we performed OCT capturing, as the retinochoroidal layers were positioned inferiorly on the scans to acquire maximum imaging depth into the vitreous. Thus, the possibility of misjudgment is minimized.

Third, the single B-scan is relatively a rough diagnosis, and it is insufficient to elucidate the entire vitreous anatomy. Therefore, a more comprehensive study is required to clarify the vitreous change with aging. Furthermore, a study of volumetric analysis of anterior human vitreous revealed the microstructural features showing the quantitative association of vitreous indices with aging^20^. Cube and radial scan modes may be helpful for the three-dimensional analysis of PVD status, although these scans required more time to produce the same image quality as that of the single B-scan. Future scientific advancement may enable high-resolution volumetric evaluation of posterior vitreous in feasible examination time.

Fourth, although age, sex, and refraction have been reported to be associated with PVD^21–23^, the sex ratios and refractions differed among the age groups in this study. However, in further analysis, there were no significant differences in the mean age between males and females and in the PVD stage between both sexes. In addition, although refractive power can influence the actual scan size, only subjects with a refractive power of + 3.0 to − 5.0 diopters were included in this study. Thus, we believe that the effects of sex and refractive power were minimized. Finally, 10.1% of the subjects had pseudophakic eyes. Studies using SD-OCT have shown that cataract surgery accelerates PVD^24^. To more accurately assess the natural history of PVD, we need to evaluate only phakic eyes, even in the elderly population.

Despite these limitations, we minimized the influence of individual characteristics and enrolled a large sample size. Importantly, we also evaluated PVD quantitatively. In previous OCT studies, PVD was qualitatively assessed. Further, paramacular and perifoveal PVDs were defined as stages 1 and 2, respectively; thus, limiting the reproducibility of the PVD staging system. Meanwhile, the staging system used in this study can be conveniently applied to staging across different OCT devices and graders; thus, making it a suitable method in future OCT studies for PVD.

In conclusion, partial PVD in eyes without pathologies occurs at an earlier age than previously reported, as evidenced by the detection of PVD even in subjects aged < 10 years. The PVD stage progresses with age, but the rate is concordant between the left and right eyes. These findings can be useful for future widefield OCT studies on vitreoretinal interface diseases.

## Methods

### Study design and subjects

This was a prospective observational cross-sectional study of healthy subjects enrolled between March 2020 and January 2021 at Shinshu University. Enrollment was only terminated when each age group involved at least 20 subjects. The exclusion criteria were (1) present or past vitreous-retina-choroid disease, (2) hyperopia > + 3.0 diopters or myopia exceeding − 5.0 diopters, (3) history of eye operation except for cataract surgery, and (4) ocular media opacity that may affect the image quality in either eye.

This study was approved by the Institutional Review Board of Shinshu University School of Medicine (approval number 4908) and adhered to the tenets set forth in the Declaration of Helsinki. Written informed consent was obtained from all the subjects or the parent/guardian for subjects younger than 20 years.

### OCT scans and PVD staging

All OCT examinations were performed with 23-mm scans, corresponding to a 78° angle of view, using a commercially available SS-OCT (Xephilio OCT-S1, Canon, Tokyo, Japan). Standardized horizontal vitreoretinal sections through the fovea were collected for the bilateral eyes in each subject.

**Figure 2:**
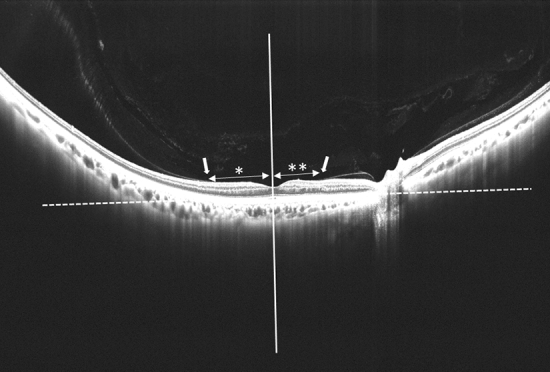
Quantitative evaluation of the point of vitreous detachment perpendicular line to the macula (PLM) indicated by a white solid line is assumed to pass the minimum distance from the center of the fovea to the choroidal line indicated by a white dot line. This is a tangent drawn to the outer border of the retinal pigment epithelium at the bottom of the foveal center. The distance of PLM to the point of vitreous detachment (white arrow) (PLM-VD) is measured for PVD staging. In this case, the temporal PLM VD (*) is 2568 µm, and the nasal PLM VD (**) is 1802 µm, both larger than 750 µm; therefore, the diagnosis is PVD stage 1.

To visualize the vitreoretinal interface in more detail, all SS-OCT scans were obtained by decreasing subtracting 3 diopters from the automated focusing of the internal ref of SS-OCT^25^. The refraction values were also measured with a refractometer built into the device. After importing the SS-OCT images into Image J software (National Institutes of Health, Bethesda, Maryland, USA), the brightness and contrast were adjusted by grader preference. The point of vitreous detachment, if present, was recorded according to its coordinates in a grid chart. The perpendicular line to the macula (PLM) was assumed to pass the minimum distance from the center of the fovea to the choroidal line. The distance from the PLM to the point of vitreous detachment (PLM-VD) was measured for PVD staging (Fig. 2).

**Figure 3:**
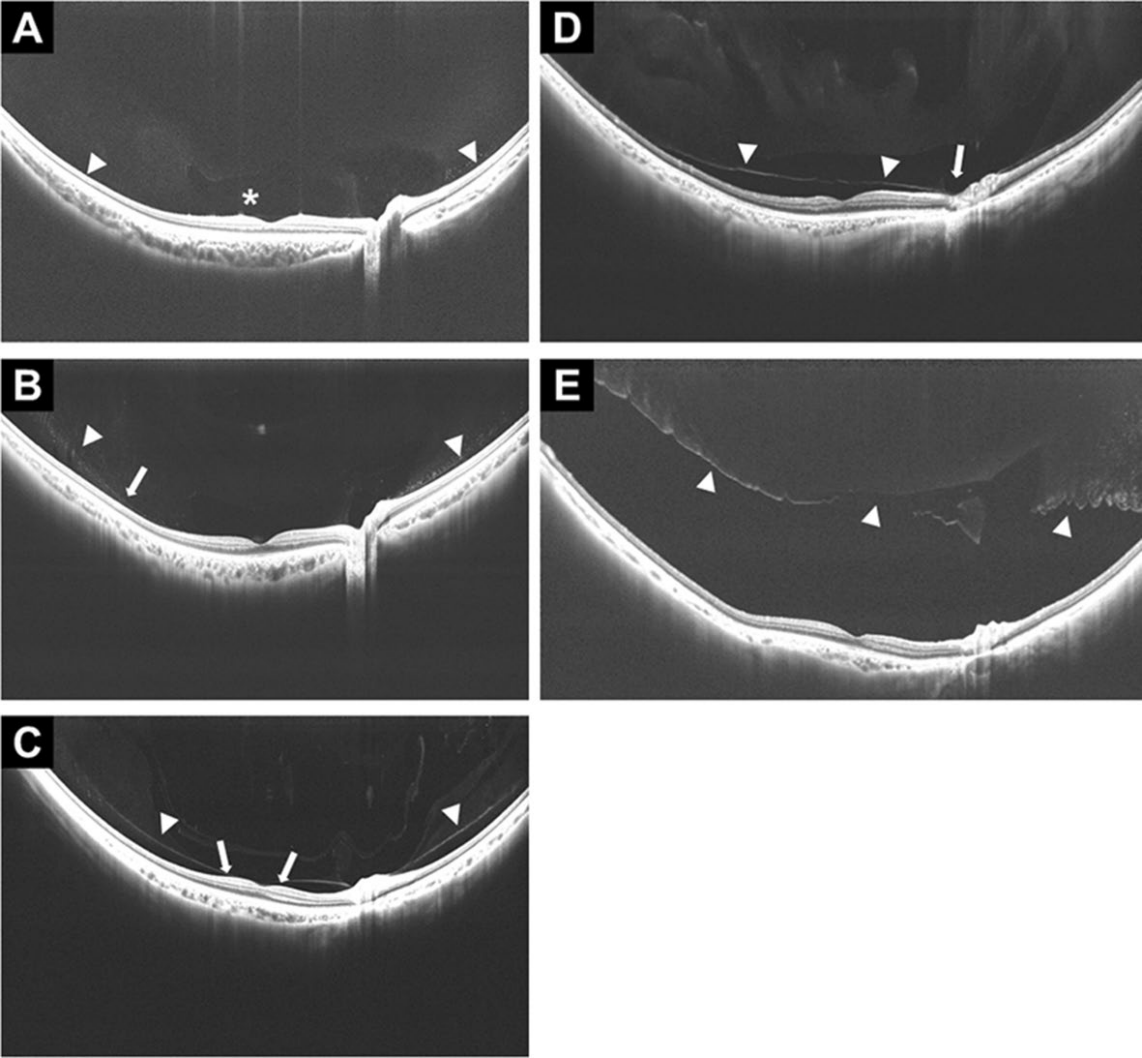
PVD stages observed on widefield SS-OCT. The right eye of a 5-year-old girl with stage 0 PVD shows the paramacular bursa (asterisk) without separation of the posterior vitreous cortex and the retina (**A**). The right eye of a 23-year-old man with stage 1 PVD shows the partial separation of the posterior vitreous cortex that did not extend into the fovea (arrow) (PLM-VD of 4876 µm (≥ 750 µm) (**B**). The right eye of a 47-year-old man with stage 2 PVD shows vitreoretinal separation from a part of the fovea with persistent attachment to the foveola (arrows). This is defined as a shorter (nasal) PLM-VD of 136 µm (< 750 µm) (**C**). The right eye of a 69-year-old man with stage 3 PVD shows separation from the entire macula with persistent attachment to the optic nerve (arrow) (**D**). The right eye of an 88-year-old woman with stage 4 shows no adhesion between the retina and the posterior vitreous cortex. Arrowheads in all images indicate the posterior vitreous (**E**).

PVD staging was independently performed by two experienced retina physicians (Y.C, T.H) by reviewing all 23-mm SS-OCT images. Staging was according to a modified version of a previously described system^6^, and disagreements were resolved via discussions. The PVD stage was divided into five categories as follows (Fig. 3). Stage 0 was defined as no PVD, that is, a visualization of the paramacular bursa without separation of the posterior vitreous cortex and the retina. Stage 1 was defined as peripheral PVD limited to paramacular, peripheral, or from paramacular to peripheral zones, that is, partial separation of the posterior vitreous cortex that did not extend into the fovea, which was considered to be the central 1500 μm diameter of the macula. Therefore, stage 1 was characterized as PLM-VD of ≥ 750 µm radius of the fovea. Stage 2 was defined as perifoveal PVD expanding to the periphery, that is, vitreoretinal separation from a part of the fovea with persistent attachment to the foveola, which was characterized as PLM-VD < 750 µm. Stage 3 was defined as peripapillary PVD with persistent vitreopapillary adhesion alone, that is, a separation from the entire macula with persistent attachment to the optic nerve. Stage 4 was defined as complete PVD, that is, no adhesion between the retina and the posterior vitreous cortex.

### Statistical analysis

Categorical variables were presented as numbers and percentages and analyzed using the chi-square tests. Continuous variables were expressed as the mean values ± standard deviation and analyzed using paired t-tests or the Mann–Whitney U test. The correlations between age and PVD stage were investigated using Spearman’s correlation coefficient. The Kruskal–Wallis rank-sum test was used to compare the PVD stages for bilateral eyes. All statistical analyses were performed using SPSS V.24.0 (IBM, Armonk, New York, USA). A *P* value of < 0.05 was considered statistically significant.

### Meeting presentation

The 125th Annual meeting of the Japanese Ophthalmological Society. Osaka, Japan, 2021.

## Supplementary Information

Supplementary Figure 1.

Supplementary Figure 2.

## Abbreviations

PLM: Perpendicular line to the macula
PLM-VD: Distance from the PLM to the point of vitreous detachment
PVD: Posterior vitreous detachment
SD-OCT: Spectral-domain optical coherence tomography
SS-OCT: Swept-source optical coherence tomography
