# Supplementary figures and images for: Evaluating posterior vitreous detachment by widefield 23-mm swept-source optical coherence tomography imaging in healthy subjects
Source: Sci Rep. 2021 Oct 5;11:19754. doi: 10.1038/s41598-021-99372-z (PMC8492648; doi:10.1038/s41598-021-99372-z)

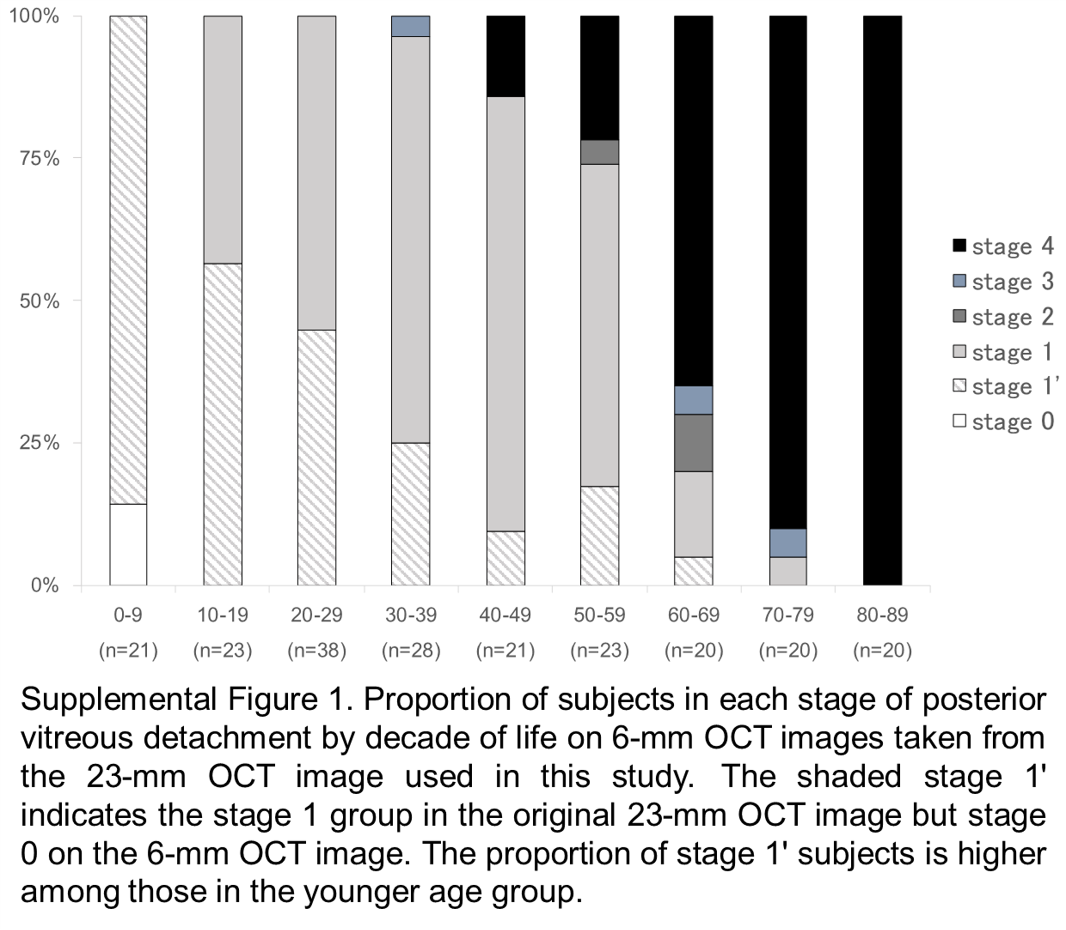

Supplement: Supplementary file 1 — Supplementary Figure 1. [file 41598_2021_99372_MOESM1_ESM.tif]

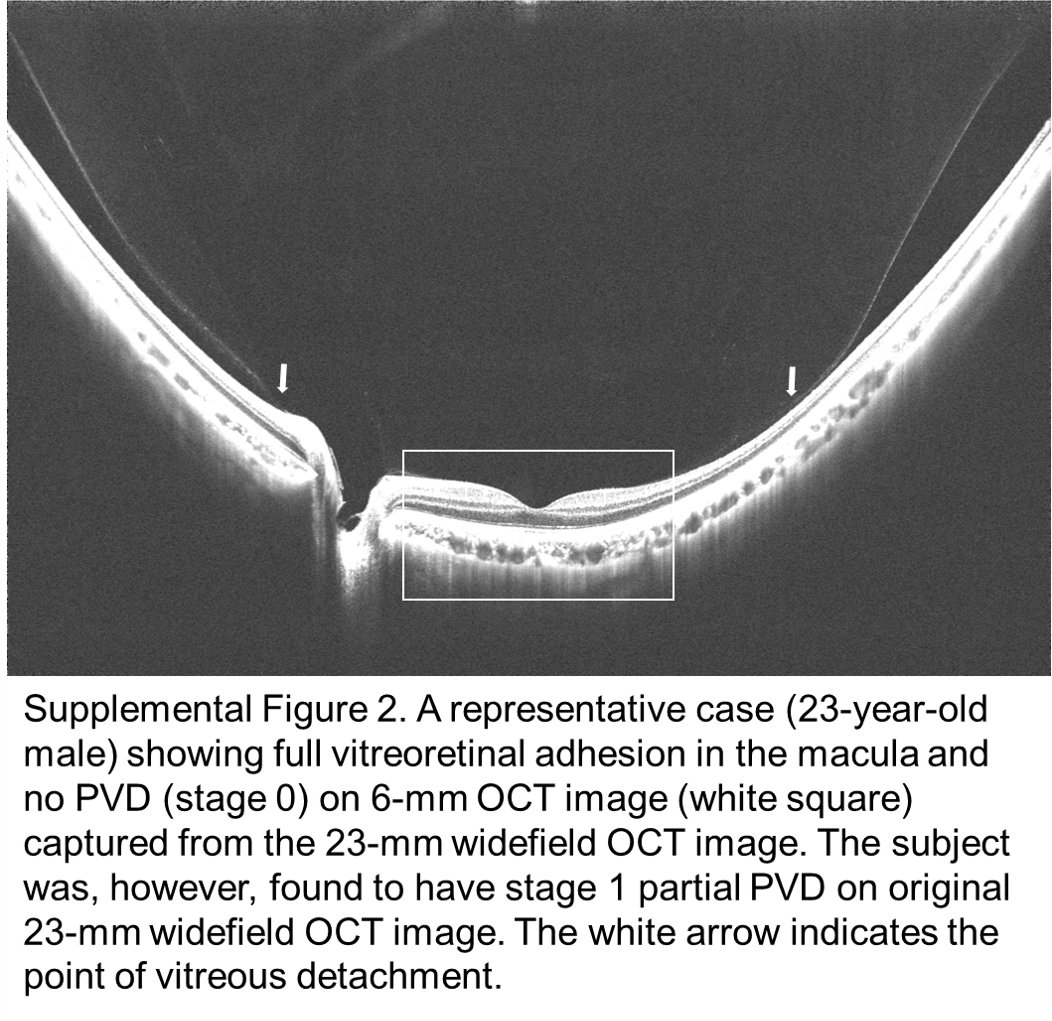

Supplement: Supplementary file 2 — Supplementary Figure 2. [file 41598_2021_99372_MOESM2_ESM.tif]
